# Percutaneous sclerotherapy for venous malformations in the extremities: clinical outcomes and predictors of patient satisfaction

**DOI:** 10.1186/2193-1801-3-520

**Published:** 2014-09-11

**Authors:** Masahisa Nakamura, Keigo Osuga, Noboru Maeda, Hiroki Higashihara, Kenichiro Hamada, Nobuyuki Hashimoto, Shuichiro Uehara, Noriyuki Tomiyama

**Affiliations:** Department of Diagnostic and Interventional Radiology, Osaka University Graduate School of Medicine, 2-2 Yamadaoka Suita, Osaka, 565-0871 Japan; Department of Orthopedic Surgery, Osaka University Graduate School of Medicine, 2-2 Yamadaoka Suita, Osaka, 565-0871 Japan; Department of Orthopedic Surgery, Osaka Medical Center for Cancer and Cardiovascular Diseases, 1-3-3 Nakmichi Higashinari, Osaka, 537-8511 Japan; Department of Pediatric Surgery, Osaka University Graduate School of Medicine, 2-2 Yamadaoka Suita, Osaka, 565-0871 Japan

**Keywords:** Venous malformation, Sclerotherapy, Extremity, Adjacent bone change, Predictor

## Abstract

The purpose of this study is to retrospectively evaluate the clinical outcomes and to identify the predictors ofpatient satisfaction after percutaneous sclerotherapy for venous malformations (VMs) in the extremities. A totalof 48 patients with VMs in the extremities over 10 years of age underwent sclerotherapy to relieve symptoms, such as pain, swelling, functional limitations, and cosmetic problems. Self-assessment questionnaires were sentto rate the degree of symptom improvement and level of satisfaction. Clinical and imaging data from medicalrecords were analyzed to obtain information about VMs and sclerotherapy. The predictors for patientsatisfaction were determined by univariate and multivariate analysis of clinical variables. Forty patients (meanage, 28.2 years; range, 11-69 years) responded to the survey. Sixteen patients had VMs in the upper extremities, and 24 patients had VMs in the lower extremities. In 12 patients (30%), adjacent bone change was seen. After amean of 2.6 (range 1-10) sclerotherapy sessions, good response to pain, swelling, dysfunction, and cosmeticproblems was obtained in 83%, 74%, 79%, and 50% of patients, respectively. Thirty-two patients (80%) weresatisfied with their outcomes. On univariate analysis, absence of adjacent bone change, maximum diameter (<6.7 cm), and number of sclerotherapy sessions (<3) were significantly associated with patient satisfaction.Multivariate analysis revealed absence of adjacent bone change (odds ratio, 7.56; 95% confidence interval, 1.02-55.8) as an independent predictor for satisfaction. Thus, adjacent bone change significantly portended adissatisfied patient. In conclusion, percutaneous sclerotherapy was effective to relieve symptoms of VMs in theextremities, and most patients were satisfied with the outcomes. However, adjacent bone change was asignificant predictor of patient dissatisfaction.

## Introduction

Venous malformations (VMs) are the most common type of vascular malformations. VMs are comprised of dilated, thin walled, sponge-like abnormal channels with deficient smooth muscle (Mulliken & Glowacki 1982). They are located in any portion of the body, and the main locations are the extremities (40%), the head and neck (40%), and the trunk (20%) (Dubois & Garel 1999). VMs in the extremities are sometimes asymptomatic but often present with various symptoms of pain, swelling, functional limitations, cosmetic disfigurements, and so on (Mendonca et al. 2010). Surgery, sclerotherapy, laser therapy, and conservative treatments such as elastic compression garments have been used for the management of VMs (Van der Vleuten et al. 2014). Sclerotherapy has also played a central role as a minimally invasive and effective treatment (Van der Vleuten et al. 2014; Berenguer et al. 1999; Tan et al. 2007).

Not all the outcomes of sclerotherapy for VMs have been satisfying, however, and overly aggressive treatment can make the condition worse rather than improve it and result in serious complications (Lee et al. 2008). A few studies (Berenguer et al. 1999; Yun et al. 2009) have evaluated predictors of response after sclerotherapy by multivariate analysis. Identification of response predictors is clinically useful to help guide patient selection and might thereby help improve treatment results and minimize complications. The clinical manifestations of VMs vary according to anatomic locations. However, predictors for response to sclerotherapy for extremities VMs have not yet been identified. The aim of this study was to evaluate clinical outcomes and predictors for patient satisfaction after sclerotherapy for VMs in the extremities.

## Materials and methods

### Patients

Following approval from the Institutional Review Board, we performed a retrospective study of a clinical database for 128 patients treated with sclerotherapy in our department between December 2002 and May 2012. The inclusion criteria for the present study population were: patients over 10 years of age who had undergone sclerotherapy for VMs in the extremities; the sclerotherapy treatment was considered to be finished; and more than 6 months had passed since the last treatment. Patients with combined vascular malformations (e.g., capillary VMs, lymphatic VMs, capillary-lymphatic VMs, Klippel-Trenauney syndrome) were excluded. Six patients who underwent surgical resection after sclerotherapy were also excluded.

VMs were diagnosed by a combination of clinical examination and noninvasive studies, such as magnetic resonance imaging (MRI), duplex ultrasonography (US), and plain film radiography, and were confirmed by fluoroscopic imaging using direct puncture. The treatment modality was determined by a multidisciplinary team in our vascular malformation clinic, involving interventional radiologists, plastic surgeons, orthopedists, pediatric surgeons, dermatologists, and pathologists. The indications for invasive treatment included worsening pain, increased swelling, reduced function, and severe cosmetic disfigurement, based on balance between the degree of symptoms and the risk of intervention.

Among 128 patients treated with sclerotherapy, 48 patients who met the inclusion criteria were contacted by telephone and were sent a questionnaire. Forty patients who submitted self-assessment data were included in the study.

### Procedures

After proper counseling and after obtaining written informed consent from patients/parents, treatment of VMs was performed using direct percutaneous injection of 3% polidocanol, absolute ethanol, or 5% ethanolamine oleate (EO). Treatment for VMs was typically tailored to each lesion and to each patient; therefore, it was not possible to utilize a uniform treatment protocol. As sclerosants, 3% polidocanol foam was mainly used. When polidocanol was not effective, we tended to use ethanol or EO. General anesthesia was used when performing ethanol injection. Otherwise, conscious sedation and local anesthesia were chosen for pain control.

Direct puncture of the lesion was performed using a 21- to 27-gauge needle under ultrasound guidance or by direct observation. Multiple punctures were performed to inject sclerosant into the majority of the lesion. The volume injected was based on the patient’s weight and on the size of VM. The maximum dose of polidocanol, ethanol, and EO injected per person was 10 ml, 0.4 ml/kg, and 0.4 ml/kg, respectively.

**Figure 1:**
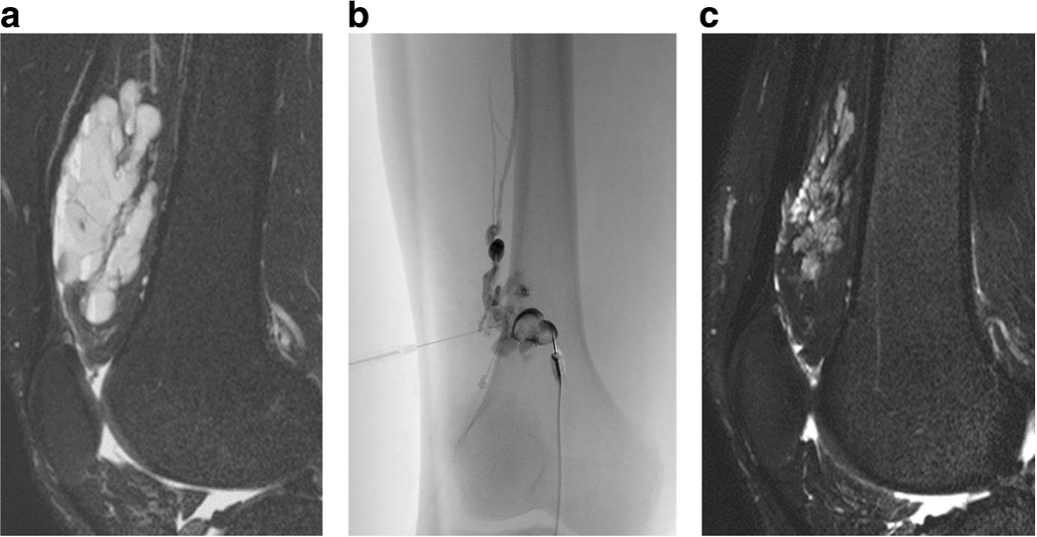
**A 30-year-old-male with pain, swelling, and dysfunction of the right knee joint. A**. The sagittal fat-saturated T2-weighted MR image before treatment shows a lobulated high-intensity mass in the suprapatellar bursa. **B**. Direct puncture phlebography shows the lesion cavity and the conducting vein (Type 2). Sclerotherapy was performed with 3% polidocanol foam. **C**. After two sessions, MR imaging shows a decrease in size and signal intensity of the mass. The patient had improvement of symptoms and indicated satisfaction on the questionnaire.

The decision to perform repeat sclerotherapy was based on a discussion with the patient. The goal of treatment was not to eliminate the lesion, but rather to improve symptoms. Thus, even if the lesion persistent, treatment was discontinued if those goals were achieved (Figures 1).

Complications were classified into major or minor complications, according to Society of Interventional Radiology reporting standards (Omary et al. 2003). Major complications were those that resulted in an unplanned increase in the level of care, permanent adverse sequelae, or death. Minor complications were those that resulted in no sequelae with or without nominal therapy requirement.

### Questionnaire

A self-assessment questionnaire was sent to the patients in December 2012. The questionnaire contained items assessing symptoms and satisfaction levels. In the questionnaire, patients were asked for specific symptoms (e.g., pain, swelling, functional limitations, and cosmetic disfigurements), and a four-point scale was used to rate the degree of symptom improvement as follows: markedly improved, moderately improved, no change, and worsening (van der Linden et al. 2009). Similarly, patients were asked whether they were satisfied with sclerotherapy as follows: very satisfied, satisfied, dissatisfied, or neither. “Markedly improved” and “improved” were defined as a “good response”, and “very satisfied” and “satisfied” were defined as “satisfaction”.

### Clinical variables

Data with regard to patient demographics, clinical assessments, imaging studies, treatments, and treatment complications were obtained from the medical charts and imaging, as collected by two of the authors (MN and KO). All patients underwent pre-MRI. Clinical variables included demographic (sex), and imaging variables (VM location, VM size, VM margin, adjacent bone change, and anatomical pattern of draining veins on direct puncture venography), and procedure variables (the number of sclerotherapy, and sclerosants). Parameters that were proposed as predictors of outcomes in previous studies were evaluated in the present study (Berenguer et al. 1999; Puig et al. 2003; Yun et al. 2009; Jin et al. 2009; Mimura et al. 2009; Mendonca et al. 2010). Although adjacent bone changes, such as periosteal reaction, cortical irregularity including thickening or erosion, and medullary signal change, are often seen in VMs involving deep tissues (Ly et al. 2003), this change has not been evaluated a predictors of outcomes in previous reports. Thus, we studied whether “adjacent bone change” on MRI and plain film radiography was a predictor of outcomes in our study. The diameters of lesions were measured using MR images. Based on MRI, VMs were categorized into two margin types: a well-defined margin was defined as a sharp transition from surrounding tissue (n = 17), whereas an ill-defined margin was defined as an irregular interface with surrounding tissue (n = 23) (Jin et al. 2009). With regard to anatomical pattern of draining veins on direct puncture venography, we classified VMs into to the four types; Type 1 – isolated malformation without peripheral drainage, Type 2 – malformation that drains into normal veins, Type 3 – malformation that drains into dysplastic veins, Type 4 – malformation that represents a dysplasia (Puig et al. 2003). In our study, no lesions of Type 4 were included.

### Statistical analysis

We evaluated predictors of patient satisfaction, performing uni- and multivariate analysis of the clinical variables. The cut-off score for patient age, VM size, and the number of sclerotherapy treatments were determined by receiver operating characteristic (ROC) curve analysis. Univariate analysis was performed to compare variables between the “satisfaction” group and the “non-satisfaction” group using the chi-square test and the Kruskal-wallis test. For multivariate analysis, a binary logistic regression model was used to identify independent predictors. P values of less than 0.05 were considered to indicate statistical significance. Statistical analysis was performed using SPSS Statistics 21 software (IBM Corporation, USA).

## Results

Patient demographics and clinical data were summarized in Table 1. Distribution of the lesions in the extremities was given in Table 2. A total of 105 treatment sessions were performed (mean, 2.6 sessions per patient; range, 1–10 sessions). The mean number of punctures per session was 5.9 (range, 1–27). The sclerosants used for treatment are listed in Table 1. Polidocanol was used in a majority (37 of 40) of patients. In five patients, pneumatic cuff tourniquets were used beyond the lesion’s venous outflow. The mean follow-up period was 2.3 years (range, 7 months-7.5 years).

**Figure 2:**
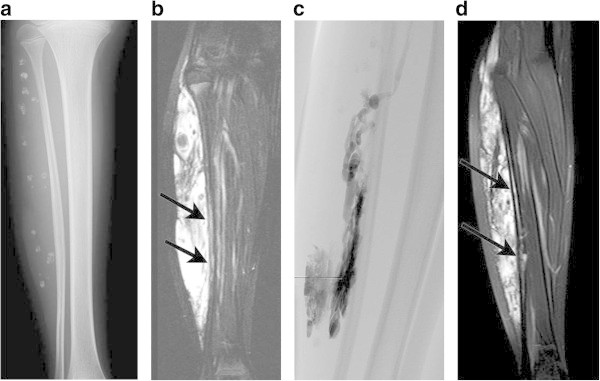
**A 12-year-old-female with symptoms of pain and swelling of the right calf. A**. Radiograph of the right calf demonstrates phleboliths and cortical irregularity in the fibula. **B**. The coronal fat-saturated T2-weighted MR image before treatment shows a large and infiltrating mass adjacent to the fibula with diffuse marrow signal abnormality (arrows). **C**. Direct puncture phlebography reveals VM with dysmorphic veins and early venous return of the peroneal vein (Type 3). After sclerotherapy with ethanol, the patient had onset of temporary peroneal nerve paralysis. **D**. Although the lesion shows a decrease in thickness and signal intensity on MRI, the marrow signal abnormality (arrows) remains unchanged. She reported no change in symptoms and indicated dissatisfaction on the questionnaire.

Two major complications occurred after sclerotherapy. One patient treated with 10.5 ml of absolute ethanol had peroneal nerve paralysis for 9 months (Figures 2). The other patient treated with 20 ml EO had acute renal failure and needed temporary hemodialysis. Minor complications like local swelling and pain were experienced in most cases for a few days and were well controlled with NSAIDs.

**Table 1:** **Patient demographics and clinical data**

Variables	n = 40
Age^a^	28.2(11–69)
Sex (male:female)	11:29
Location of VM^b^	
Upper extremity	16(40)
Lower extremity	24(60)
Previous treatment^b^	
Operation	11(28)
with sclerotherapy	2(5)
with TAE	1(3)
Number of sclerotherapy treatments^a^	2.6(1–10)
Sclerosants (partially overlapped)^b^	
Polidocanol	37(93)
Absolute ethanol	11(28)
Ethanolamine oleate	6(15)
Dose of sclerosants (ml)/session^a^	
Polidocanol	2.8(0.4-7.0)
Absolute ethanol	7.4(4.0-13)
Ethanolamine oleate	11.1(4.5-20)

^a^Data are means. Numbers in parentheses are the range.

^b^Data represent number (percentages) of patients.

**Table 2:** **Distribution of the lesions in the extremities**

Sites		N	Total
Upper extremity			16
	Shoulder	2	
	Upper arm	4	
	Elbow	3	
	Forearm	1	
	Hand	4	
	Multiple	2	
Lower extremity			24
	Buttock	2	
	Upper leg	6	
	Knee	2	
	Lower leg	6	
	Foot	7	
	Multiple	1	
Total			40

Patient self-assessment questionnaire results are given in Table 3. Before treatment, 36 of the 40 patients (90%) had disabling pain, 34 (85%) had swelling, 24 (60%) had functional limitation, and 16 (40%) had cosmetic disfigurement. Patients reported “good response” in pain (83%; 30/36), swelling (74%; 25/34), functional limitation (80%; 19/24), and cosmetic disfigurement (50%; 8/16). “Markedly improved” was noted in at least one category in 48% (19 of 40) of patients. None of the patients responded “worse” for any symptoms. In addition, 32 of 40 patients (80%) reported being “very satisfied” or “satisfied” with the treatment; these patients were defined as the “satisfaction” group.

**Table 3:** **Clinical outcomes and degree of satisfaction after sclerotherapy**

Symptom	Marked improvement	Improvement	No change	Worse	Total
Pain	13(36)	17(47)	6(17)	0	36
Swelling	11(32)	14(41)	9(26)	0	34
Functional limitations	10(42)	9(38)	5(20)	0	24
Cosmetic disfigurements	3(19)	5(31)	8(50)	0	16
	Very satisfied	Satisfied	Neither	Dissatisfied	Total
Satisfaction	16(40)	16(40)	4(10)	4(10)	40

Note. Numbers in parentheses are percentages.

On univariate analysis of variables to predict satisfaction with sclerotherapy, absence of adjacent bone change, maximum diameter (<6.7 cm), and number of sclerotherapy sessions (<3) were significantly associated with patient satisfaction (Table 4).

**Table 4:** **Univariate analysis of variables to predict satisfaction with sclerotherapy**

Variables	Satisfaction	Non-satisfaction	P value
*Patient demographics*			
Sex			0.051
Male	11	0	
Female	21	8	
Location of VM			0.333
Upper extremity	14	2	
Lower extremity	18	6	
*Imaging variables*			
Maximum diameter of VM			0.018
<6.7 cm	19	1	
≥6.7 cm	13	7	
Margin on MRI			0.055
Limited	16	1	
Infiltrating	16	7	
Adjacent bone change			0.002
Absent	26	2	
Present	6	6	
Anatomical pattern of draining veins			0.361
Type 1	19	3	
Type 2	8	2	
Type 3	5	3	
*Procedure variables*			
Session number of sclerotherapy^a^			0.014
<3	23	2	
≥3	9	6	
Sclerosants			0.32
Polidocanol only	22	4	
Other	10	4	

^a^The Kruskal-wallis test.

**Figure 3:**
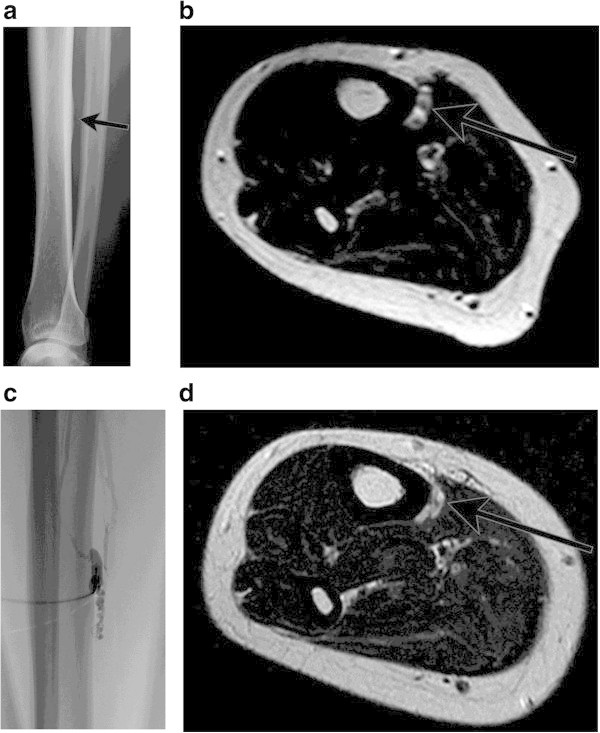
**A 37-year-old-female with symptoms of severe pain of the right lower leg. A**. Radiograph of the right lower leg demonstrates tiny periosteal reaction in the tibia (arrows). **B**. The axial T2-weighted MR image before treatment shows a tiny lesion adjacent to the tibia with cortical irregularity (arrows). **C**. Direct puncture phlebography reveals VM with normal veins and early venous return (Type 2). Sclerotherapy was performed with 3% polidocanol foam. **D**. Although the lesion decreased in size and signal intensity on MRI (arrows), she answered no change in symptoms and dissatisfaction on the questionnaire.

Table 5 shows the result of multivariate analysis. Absence of adjacent bone change (odds ratio, 7.56; 95% confidence interval, 1.02-55.8) was the only independent predictor of patient satisfaction. Among 27 (68%) patients with VMs adjacent to the bones, 12 patients (30%) showed the adjacent bone change (Figures 3).

**Table 5:** **Multivariate analysis of variables to predict satisfaction with sclerotherapy**

Variables	P value	Odds ratio	95% confidence interval
Absence of adjacent bone changes	0.048	7.56	1.02-55.8
Maximum diameter (<6.7 cm)	0.308	3.70	.299-45.8
Session number of sclerotherapy (<3)	0.240	3.56	.429-25.5

## Discussion

A comprehensive classification of vascular anomalies was accepted by the International Society for the Study of Vascular Anomalies (ISSVA) in 1996 (Enjolras & Mulliken 1997). Two major categories of lesions emerged: vascular tumors and vascular malformations. Differentiating between vascular tumors and malformations is essential, as their clinical, radiological and pathologic features differ. Further, their associated morbidity and their management are quite different. VMs are low-flow vascular malformations and can infiltrate skin, muscles, joints, and sometimes bones. In 2013, a “modified” Hamburg classification was adopted to emphasize the importance of extratruncular vs. truncular sub-types of VMs; ISSVA Classification was reinforced with an additional review on syndrome-based classification (Lee et al. 2014). The new classification incorporated the embryological origin, morphological differences, unique characteristics, prognosis and recurrence rates of VMs based on this embryological classification.

Symptoms are dependent on the anatomic location of the lesion. Pain and swelling are common symptoms associated with all VMs. With craniofacial lesions, cosmetic disfigurement may be more debilitating than functional limitations (Lee & Chen 2005). In contrast, the management of VMs in the extremities is often difficult due to functional problems rather than cosmetic concerns (Mendonca et al. 2010). In this series, 24 patients (60%) reported functional limitations. Thus, we focused on VMs in the extremities.

Sclerotherapy is now the primary treatment of choice for VMs. Several sclerosants have been proven effective and vary in their mode of action and relative toxicity (Van der Vleuten et al. 2014). Currently, there is no consensus as to the best sclerosant. All sclerosants are associated with potential complications, including skin necrosis, peripheral nerve injury, hemoglobinuria, thromboembolism, infection and delayed muscle fibrosis (Burrows 2013). We mainly use polidocanol, because it has sufficient effect and because it is associated with lower major complication rates (Blaise et al. 2011). Indeed, in our cohort, there were no major complications after sclerotherapy using polidocanol.

The efficacy of sclerotherapy for VMs is difficult to evaluate, as there are no standard assessment criteria (Rautio et al. 2004). We attempted to evaluate treatment outcomes according to overall patient satisfaction and subjective patient response using a self-assessment questionnaire rather than attempt to quantify morphologic response (van der Linden et al. 2009). This is because lesion sizes and symptoms of VMs vary widely and there is often discrepancy between the clinical and morphologic responses to sclerotherapy (Tan et al. 2007; Yun et al. 2009).

Our analysis of a cohort of 40 patients who responded to follow-up questionnaires showed that 80% of patients were satisfied with treatment outcome and that only two major complications (5%) occurred. Pain, swelling, and functional limitations were improved in about 80% of patients, whereas cosmetic improvement was seen in 50% of patients. Van der Vleuten et al. ( 2014) conducted a systematic review of studies investigating treatment for VMs. They reported that sclerotherapy was effective in 65% to 90% of cases. Our results are comparable to those seen in previous reports and indicate that sclerotherapy was minimally invasive and effective as a primary treatment for VMs.

Identification of predictors of response to sclerotherapy is important to optimize outcomes through appropriate patient selection. Previous reports have investigated predictors of response to sclerotherapy in VM patients. For example, Berenguer et al. ( 1999) reported that male sex and number of sclerotherapy sessions were independent predictors of good outcomes. Goyal et al. ( 2002) proposed that patients with well defined, small VMs on MRI imaging had a better response to sclerotherapy. Yun et al. ( 2009) identified no or delayed visualization of drainage veins, a well-defined margin on MRI, and female sex as predictors of good outcomes. Mimura et al. ( 2009) revealed a better therapeutic effect in patients with small VMs, well-defined VMs, and VMs with good stasis of sclerosant during sclerotherapy.

In our study, adjacent bone change, maximum diameter of VM, and number of sclerotherapy sessions were significantly associated with patient satisfaction on univariate analysis. Multivariate analysis revealed that absence of adjacent bone change was an independent predictor for good satisfaction after sclerotherapy, whereas sex, VM location, VM margin, and anatomical pattern of draining veins on a venography were not. Thus, poor outcomes are expected in VMs with adjacent bone change. Mendonca et al. ( 2010) estimated that VMs with bone or joint involvement were associated with a higher risk of symptom recurrence. Goto et al. ( 2001) reported that hemangiomas with adjacent periosteal new bone formation were more painful than those without it. These results support our findings.

Bone changes adjacent to VMs (also referred to as “soft-tissue hemangiomas” in the literature) were observed in 19-63% of patients on plain film or MRI (Mendonca et al. 2010; Ly et al. 2003; Goto et al. 2001; Sung et al. 1998; Enjolras et al. 1997; Breugem et al. 2001; Pourbagher et al. 2011). In our cohort, 12 patients (30%) had bone changes adjacent to VMs. The precise mechanism of adjacent bone change remains unknown. Several factors could contribute to adjacent bone change, including physical irritation, an extrinsic pressure and passive hyperemia (Sung et al. 1998; Goto et al. 2001; Pourbagher et al. 2011). Bone homeostasis is maintained by the balance between bone resorption and formation and is affected by local oxygen tension and pH, various cytokines, and hormones (Arnett 2010). We postulate that some cytokines and the change in local oxygen tension and pH due to latent microshunts and congestion may be one of the important factors developing the adjacent bone change around VMs, but it is still no better than a conjecture.

Further investigation is needed to clarify the effect of adjacent bone change on patient symptoms that may impair patient satisfaction to sclerotherapy. Studies of local oxygen tension and pH, bone metabolic markers of osteoblast and osteoclast function, and some cytokines might be useful in this regard.

This study had several limitations. First, the study was retrospective and had a small number of patients. Further, there were no standards for treatment indication and evaluation criteria for sclerotherapy of VMs. In addition, we did not evaluate patient mental health that may affect patient satisfaction. We may take account of the use of validated quality-of-life assessment tool, such as SF-36 and the Child Health Questionnaire (CHQ).

In conclusion, percutaneous sclerotherapy was effective in relieving symptoms in patients with VMs in the extremities. Adjacent bone change was a significant predictor of patient dissatisfaction.
